# *In silico* structural analysis of *Oryza sativa* RAD51 reveals key interactions for nucleoprotein filament assembly and regulation

**DOI:** 10.1371/journal.pone.0335974

**Published:** 2025-11-12

**Authors:** Ayesha Azeem, Syed Farhat Ali, Rana Salman Anjum

**Affiliations:** 1 KAM School of Life Sciences, Forman Christian College (A Chartered University), Lahore, Pakistan; 2 Department of Oral Oncology, Roswell Park Comprehensive Cancer Center, Buffalo, New York, United States of America; NMIMS Deemed to be University - Mumbai Campus: NMIMS, INDIA

## Abstract

Radiation sensitivity 51 (RAD51) is important for homologous recombination and DNA repair. The interaction between BRCA2 and RAD51 is crucial for the successful repair of DNA double strand breaks by homologous recombination. In the present study, through *in silico* analysis, we structurally characterized *Os*RAD51, a eukaryotic RAD51 ortholog from *Oryza sativa* Japonica A1 cultivar. Multiple sequence alignment showed the presence of conserved amino acids at the ATP- and DNA-binding sites. Several phosphorylation and ubiquitination sites were also predicted in *Os*RAD51 indicating its regulation by post-translational modifications. Structural modelling of *Os*RAD51 revealed two important regions at the protomer interface – one near the ATP-binding site (Walker A motif) and the other comprising of mainly hydrophobic residues. Polar and charge-charge interactions were noticeable at DNA-*Os*RAD51 interface of the modelled nucleoprotein filament. RAD51 assembly into the nucleoprotein filament is regulated by BRCA2. To study this interaction, *Os*RAD51 was modelled with *O. sativa* BRC repeats (*Os*BRC). *Os*BRC was found to interact with *Os*RAD51 via hydrophobic and polar interactions. Moreover, structural analysis revealed that *Os*BRC interaction site overlap with the hydrophobic pockets of *Os*RAD51 required for protomer-protomer interaction, thus regulating the assembly of *Os*RAD51 into nucleoprotein filament. *O. sativa* BRCA2 (*Os*BRCA2) was found to contain 8 BRC repeats. *Os*BRC repeats, similar to *Homo sapiens* BRC4 (*Hs*BRC4), contained a conserved motif including a phenylalanine required for interaction with *Os*RAD51. So, *Os*BRCA2 can regulate the assembly of *Os*RAD51 through BRC repeats. The results of our study provide insights about structural basis of *Os*RAD51 nucleoprotein filament assembly and its regulation by BRCA2.

## Introduction

Cellular DNA is under continuous stress through endogenous or exogenous agents. To tolerate such lesions in the DNA by faulty replication or due to ionizing radiations; nature has blessed cells with repair mechanisms to rehabilitate the damage [1]. As a result of DNA double strand break (DSB), homologous recombination (HR) repair pathway is the cell’s best choice which is active especially in the S phase of the cell cycle [2]. Despite its role in DNA repair in mitotic cells, HR is involved in creating genetic variability in meiotic cells [3]. Moreover, the phenomenon of HR can be applied for genome editing and genetic modifications in plants [4].

RAD51 is the eukaryotic homologue of RecA/RadA recombinases that perform a key step in the HR pathway in all three domains of life. Common feature of such recombinases is to assemble onto the sites of DNA double strand breaks to form helical nucleoprotein filaments which are the active species for homology search and strand exchange reaction [5]. RAD51 is comprised of two globular domains with its C-terminal domain homologous to the core ATPase domain of *Escherichia coli* (*E. coli*) recombinase A (RecA) which is structurally similar to the ATPase domain of F1 ATPase. The core ATPase domain of RAD51 participates in multimerization while its N-terminus is proposed to interact with DNA to make a stable nucleoprotein filament [6]. Recombinase activity of RAD51 is inevitably dependent upon interaction with other accessory factors, central to which is the interaction with breast cancer susceptibility protein type 2 (BRCA2) [7]. Despite its variable size among different organisms, it interacts with RAD51 through evolutionarily conserved BRC repeats. BRC repeats binding to RAD51 mimic the mechanism of RAD51 multimerization to regulate its loading and unloading at ds-ssDNA junction. As a result of loss of BRCA2 function at cellular level, the sensitivity to cross linking agents increases [8,9].

On a global scale, rice is a major staple food with a worldwide production of nearly 787 million tons in 2021 [10]. Meiotic recombination is essential for reproduction and seed formation. *Oryza sativa* RAD51 (*Os*RAD51) plays an important role in homologous recombination for DNA repair [11]. In *O. sativa*, several paralogs of RecA/Rad51 are present. Among these RAD51C and RAD51D are important for regulating crossover maturation during meiosis [12,13]. *Os*RAD51 mutant rice plants showed vegetative growth but were sterile – signifying its role in meiotic recombination [14]. In rice, another ortholog *Os*RAD51D promotes HR and inhibits non-homologous interactions thus ensuring recombination during rice meiosis [15]. Likewise, a RAD51 ortholog of *Zea mays* (*Zm*RAD51C) has been reported to be critical for DSB repair and meiosis in maize with *Zm*RAD51C mutations resulting in sterility [16]. In addition to the established role in DNA recombination and repair, RAD51 can contribute to resistance against diseases in plants. It was found that overexpression *Zm*RAD51A in rice and *Arabidopsis* improved disease resistance in these plants [17]. Interaction of BRCA2 with RAD51 is important for regulating DNA recombination and repair. It has been reported that *O. sativa* BRCA2 (*Os*BRCA2) is critical for loading of *Os*RAD51 to DSB during meiosis in rice [18].

Despite the advancements in elucidating and understanding the function of *Os*RAD51, no significant structural studies have been reported. To address this aspect, we modelled the structure of *Os*RAD51, identified important structural features and interactions including formation of nucleoprotein filament and interaction with BRC repeats.

## Materials and methods

### Sequence search and multiple sequence alignment

*Homo sapiens* RAD51 (*Hs*RAD51) (Uniprot ID: Q06609) was used for a BLAST search against *O. sativa* genome. An ortholog *Os*RAD51A1 was identified (Uniprot ID: Q8SBB9) and used to perform multiple sequence alignment by using Clustal Omega.

The sequence of *Os*BRCA2 (accession number: Os01g0164800) was obtained from GeneBank [18]. The coding exons were analyzed by PHI–BLAST for the presence of BRC repeats (*Os*BRC).

### Analysis of post-translational modification

*In silico* analysis of post translational modifications (phosphorylation and ubiquitination) of *Os*RAD51 was done by using PhosphoSVM [19], MuSuiteDeep [20], NetPhos3.1 [21] and GPS 6.0 [22]. Prediction of phosphorylation was done for serine, threonine and tyrosine residues of *Os*RAD51 and lysine residues were analyzed for ubiquitination. The sites detected by at least three of the prediction programs were selected for analysis.

### Structural modelling and analysis

AlphaFold 3 was used to model the structure of *Os*RAD51 [23]. Models of monomeric *Os*RAD51 were generated using both “Use PDB template” and “Turn off template” settings. In addition, a homology model of *Os*RAD51 was also generated by MODELLER 10.5 [24] using *Hs*RAD51 as a template (PDB ID 5H1B). Five models were generated and the best model was selected using the DOPE score.

To study the formation of nucleoprotein filament, an oligomeric model of *Os*RAD51 was generated by using AlphaFold 3 with “Turn off template” setting. Six *Os*RAD51 monomers and six ATP molecules were modelled with ssDNA (5’-GTTACGATGTCAGTACGTTAG-3’). To evaluate the effect of a previously reported inhibitor [8], CAM833 (PDB ID 6TW9) was docked into *Os*RAD51 model using Autodock Vina [25]. Structural analysis and comparison was done by using ChimeraX.

## Results

### Sequence comparison of eukaryotic RAD51 proteins

**Fig 1:**
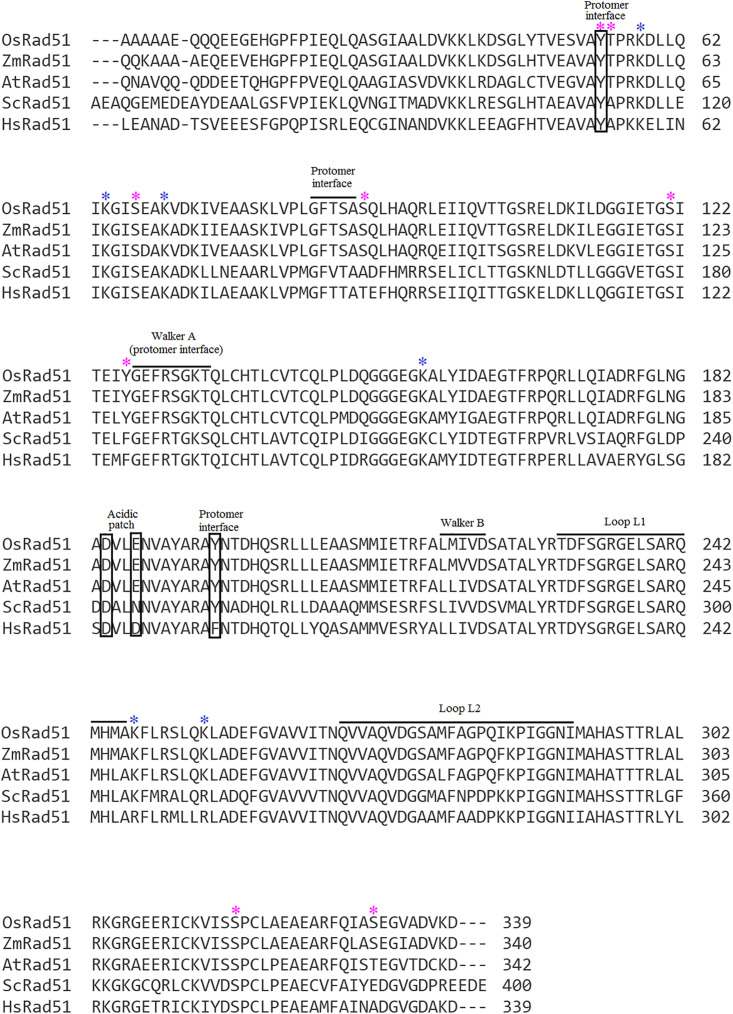
Multiple sequence alignment of RAD51 homologs showed conserved sequence features of *Os*RAD51. Protomer-protomer interfaces, Walker A and B motif, and DNA interaction loops (L1 and L2) are indicated. (*Sc*Rad51, *Saccharomyces cerevisiae* RAD51 (Uniprot ID: P25454); *Hs*Rad51, *Homo sapiens* RAD51 (Uniprot ID: Q06609); *At*Rad51, *Arabidopsis thaliana* RAD51 (Uniprot ID: P94102); *Os*Rad51, *Oryza sativa* RAD51 (Uniprot ID: Q8SBB9); *Zm*Rad51, *Zea mays* RAD51 (Uniprot ID: Q67EU8). Predicted phosphorylation and ubiquitination sites of *Os*RAD51 are indicated by magenta and blue asterisks, respectively.

In pairwise alignment, *Os*RAD51 showed high sequence similarity to other eukaryotic orthologs including *Zea mays* RAD51 (94% identity), *Arabidopsis thaliana* RAD51 (86% identity), *Saccharomyces cerevisiae* RAD51 (55% identity) and *Hs*RAD51 (69% identity) as shown in S1 Fig. In addition, multiple sequence alignment of *Os*RAD51 with human, yeast and other plant RAD51 orthologs revealed conservation of functionally important residues (Fig 1). The three main regions of *Hs*RAD51 involved in protomer-protomer association [26] are also conserved in *Os*RAD51. These include the Walker A motif (which is also involved in ATP binding), a conserved aromatic interface (Y195 and Y54) and a short motif including F86 of *Os*RAD51. The loops L1 and L2 required for DNA interaction [27] are also conserved in *Os*RAD51 and other plant RAD51 orthologs. *Os*RAD51, like its human ortholog, contained two closely placed acidic residues namely D184 and E187 (acidic patch). In *Hs*RAD51, this acidic patch is important for regulation of nucleoprotein filament assembly [28]. Taken together, these findings strongly suggest that *Os*RAD51can bind ATP and BRC repeats, and function in homologous recombination in a manner very similar to its eukaryotic counterparts (including *Hs*RAD51).

### Post translational modifications of *Os*RAD51

**Fig 2:**
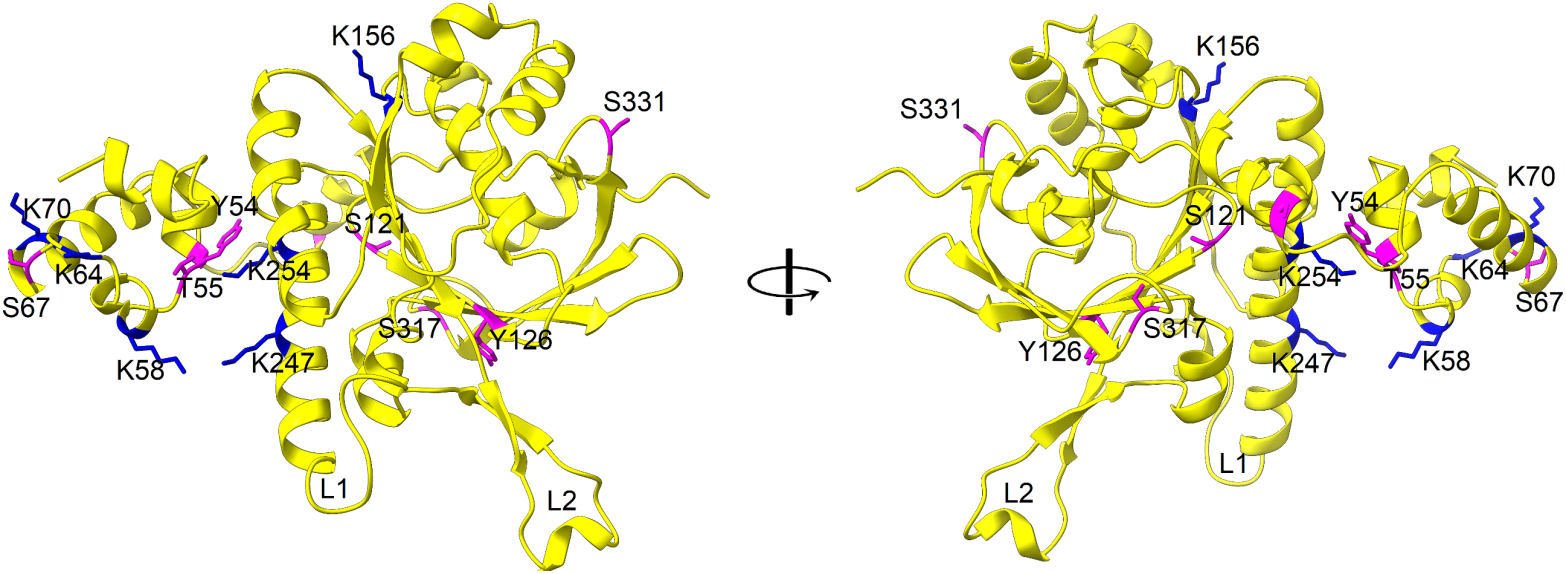
Predicted post-translational modification (PTM) sites in *Os*RAD51. *Os*RAD51 is represented as ribbon (yellow). Predicted phosphorylation sites are shown as magenta sticks and predicted ubiquitination sites as blue sticks. DNA-binding loops L1 and L2 are also shown.

RAD51 activity is regulated by post-translational modifications (PTMs) including both phosphorylation and ubiquitination [29]. So, to identify these PTMs, phosphorylation and ubiquitination sites were predicted in *Os*RAD51. It has been reported that ubiquitination of RAD51 hinders its interaction with BRCA2 [29]. Several ubiquitination sites were predicted in *Os*RAD51 including K58, K64, K70, K156, K247 and K254 (indicated by blue asterisks in Fig 1 and blue sticks in Fig 2). So, ubiquitination on these predicted sites can affect interaction of *Os*RAD51 with BRCA2 as well as its proteasome-mediated degradation [30]. Moreover, phosphorylation can also affect the activity of RAD51 [31]. Several potential phosphorylation sites were predicted in *Os*RAD51 (indicated by magenta asterisks in Fig 1 and magenta sticks in Fig 2) including Y54 (which forms the aromatic protomer-protomer interface), T55, S67, S90 (adjacent to protomer-protomer interface), S121, Y126 (adjacent to Walker A motif) S317 and S331. So, phosphorylation/dephosphorylation of these residues may influence the activity of *Os*RAD51 including ATP-binding and nucleoprotein filament assembly.

### Structural modelling and formation of nucleoprotein filament

Three types of monomeric *Os*RAD51 models were generated. Two models were generated with AlphaFold 3 using a template (“Use PDB template” setting) and without using a template (“Turn off template” setting). It was found that AlphaFold 3 selected *Hs*RAD51 (PDB ID 5JZC) as a template when “Use PDB template” setting was used. A homology model of *Os*RAD51 was also generated by using MODELLER 10.5 with *Hs*RAD51as a template. All three models were compared (S2 Fig) and backbone RMSD was calculated (S1 Table). All three models showed similar structure and domains (S2 Fig). Both AlphaFold models (using a template and without using a template) were very similar to each other with a backbone RMSD of 0.075. However, the model generated by MODELLER had a greater structural variability (with a backbone RMSD of nearly 1.0) when compared with both AlphaFold models. However, using *Hs*RAD51 as a template may bias the resulting model. So, AlphaFold *Os*RAD51 model (generated without using a template) was used for further analysis and nucleoprotein filament modeling.

**Fig 3:**
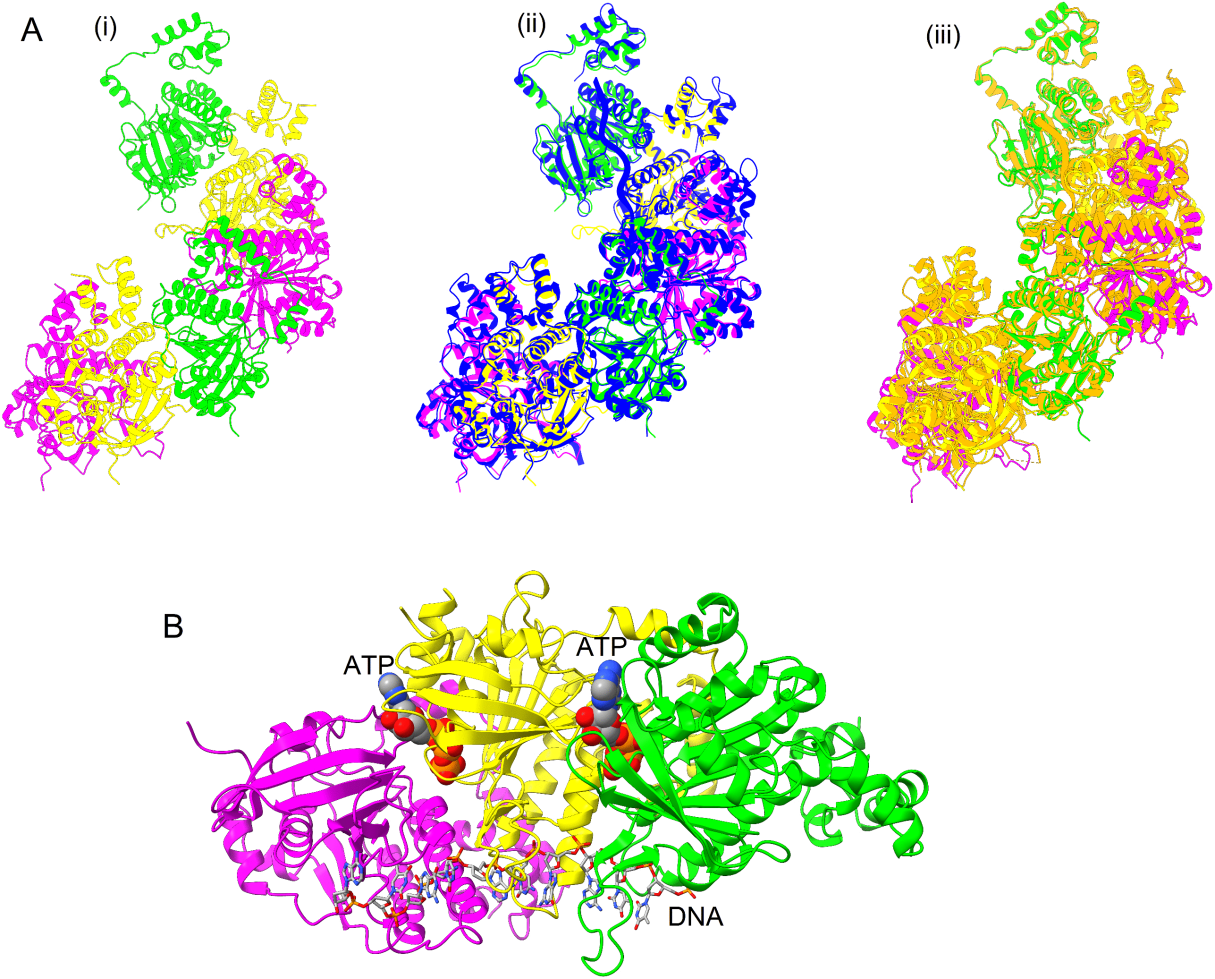
Formation of nucleoprotein filament of *Os*RAD51. (A) i: Six protomers of *Os*RAD51 nucleoprotein filament (two sets of three protomers). The repeated protomers are colored in green, yellow and magenta. ii: Superposition of *Os*RAD51 nucleoprotein filament over *Sc*RAD51 (PDB ID 9B2D, blue). iii: Superposition of *Os*RAD51 nucleoprotein filament over *Hs*RAD51 (PDB ID 5H1B, orange) (B) Overview of *Os*RAD51 nucleoprotein filament structure. The protomers are shown in same colors as in A. ATP, shown as spheres, binds at the protomer-protomer interface. Bound ssDNA is shown as sticks.

An AlphaFold model of nucleoprotein filament was generated (without using a template) with six monomers of *Os*RAD51, ssDNA and ATP (Fig 3A i). Superposition of *Os*RAD51 nucleoprotein filament model over *Sc*RAD51 (PDB ID 9B2D, Fig 3A ii, shown in blue) and *Hs*RAD51 (PDB ID 5H1B, Fig 3A iii, shown in orange) revealed significant structural similarity among the three nucleoprotein filaments.

Overall structure of *Os*RAD51 (3 protomers) with ssDNA and ATP is shown in Fig 3B. ATP is bound at the interface of the protomers with ssDNA surrounded by conserved loops L1 and L2.

### Protomer-protomer interaction

**Fig 4:**
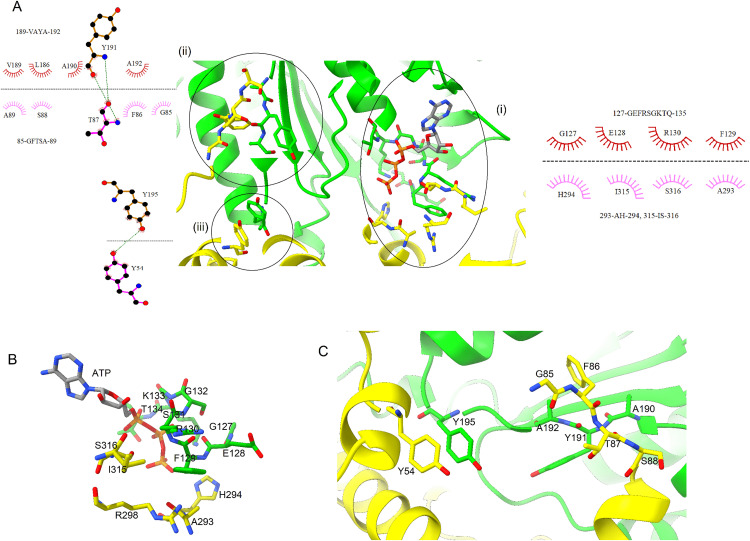
Protomer-Protomer interaction of *Os*RAD51. (A) Overview of protomer interaction. The interacting residues are shown as a 2D plot. ATP is shown as sticks at the Walker A motif. (B) Close-up view of Walker A motif interaction. (C) Hydrophobic interaction between the two protomers. (For residues shown as sticks: carbon atoms are colored according to the chain, nitrogen atoms blue and oxygen atoms red).

Overall structure of *Os*RAD51 protomer-protomer interface is shown in Fig 4A. The protomers contact each other in two distinct regions. Firstly, the Walker A motif of first subunit of *Os*RAD51 (127-GEFRSGKTQ-135) interacts with a region near loop L2 of the second subunit (including A293, H294, R298, I315 and S316) (Fig 4A i). Interestingly, these regions are highly conserved between *Hs*RAD51 and *Os*RAD51 except I315 and S316 of *Os*RAD51 which are replaced by Y315 and D316 in *Hs*RAD51. Together, these regions of the two *Os*RAD51 subunits also form the ATP binding site (Fig 4B). Secondly, a short hydrophobic region of the first protomer (190-AYA-192) assembles with a hydrophobic region (85-GFTSA-89) of the second protomer (Fig 4A ii). In addition, another aromatic interaction is found near this site. Y195 of the first protomer interacts with Y54 of the second protomer (Fig 4A iii, 4C). These conserved interactions are found among the other neighboring protomers as well.

### Interaction with ATP and DNA

**Fig 5:**
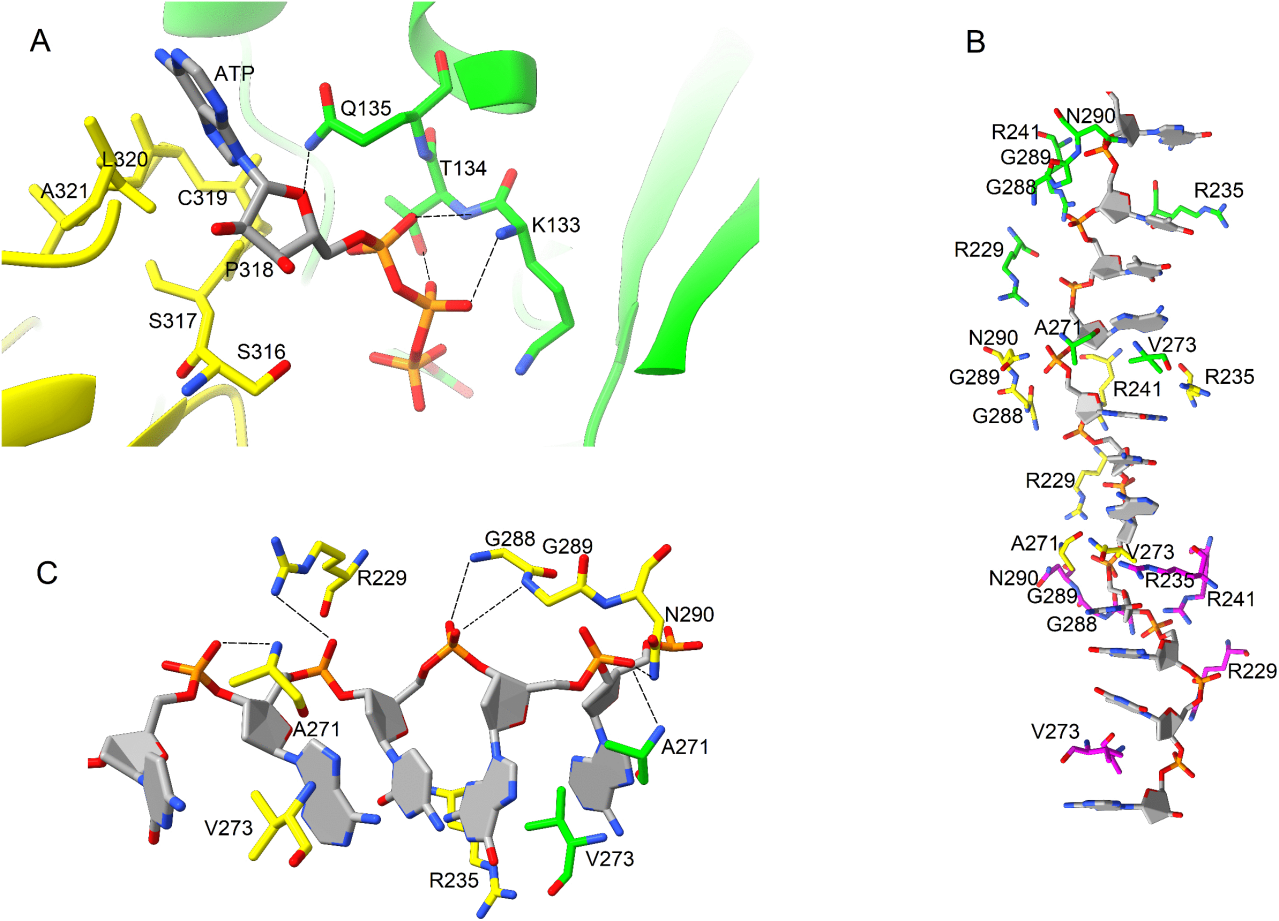
Interaction of *Os*RAD51 with ATP and DNA. (A) ATP binds at the interface of the two protomers (green and yellow) at Walker A motif. (B) Interaction of the protomers with DNA with loops L1 and L2 of each protomer surrounding the DNA. Important polar and charged amino acids of the protomers are shown. **(C)** Close-up view of *Os*RAD51 interaction with DNA triplets. Protomers interact with the backbone of DNA. Interacting residues of protomer 1 (green) and protomer 2 (yellow) are shown. (Interacting residues are marked with dashed lines. Residues represented as sticks are colored as in Fig 4).

As shown in Fig 5A, ATP is bound at the interface of the two protomers of *Os*RAD51. K133 and T134 of Walker A motif of the first protomer interact with the phosphate groups and Q135 with the ribose sugar. S315-A321 form the interacting surface of the second subunit. Moreover, *Os*RAD51 contained a conserved E163 located near AMP-PNP. In addition, D316 of *Hs*RAD51 forms a salt bridge with ATP [32]. However, in *Os*RAD51 this is replaced by S316 at the ATP binding site. Likewise, *At*RAD51 and *Zm*RAD51 also contain serine at the corresponding position (Fig 1). Collectively, these regions at the interface of the two protomers form the ATP binding site.

The loops L1 and L2 (Fig 1) are important for DNA binding and strand exchange [27]. The model of *Os*RAD51 with DNA showed that the protomers mainly interact with the backbone of ssDNA with loops L1 and L2 surrounding DNA (Fig 5B). The nucleotides of bound ssDNA interact with protomers as triplets. As represented in Fig 5C, the valine residues of the protomers separate the triplets. A271 of first protomer and N290 of the second protomer interacts with the first phosphate; G288 and G289 interact with the second phosphate; and R229 with the third phosphate of the triplet.

### Interaction with BRC repeat

**Fig 6:**
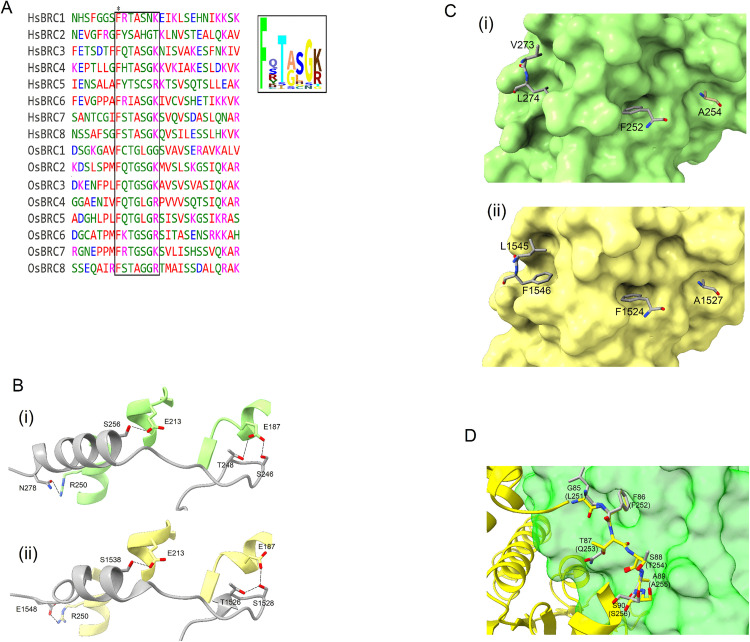
Interaction of BRC repeat with *Os*Rad51. (A) Sequence alignment of BRC repeats in *Hs*RAD51 and *Os*RAD51 shows the presence of conserved Fxx(A/G) motif. A logo of this conserved motif is shown in the inset. (B) i: Polar contacts of *Os*BRC8 (grey) with *Os*RAD51 (green). ii: Polar contacts of *Hs*BRC4 (grey) with *Hs*RAD51 (yellow, PDB ID 1N0W). (C) i: Non-polar residues of *Os*BRC8 (grey) occupy the hydrophobic pockets of *Os*RAD51 (green surface). ii: Non-polar residues of *Hs*BRC4 (grey) in the hydrophobic pockets of *Hs*RAD51 (yellow surface, PDB ID 1N0W) (D) *Os*BRC8 (grey) binds to the protomer-protomer interface of *Os*RAD51. The interacting residues of second protomer (yellow) are shown as sticks. *Os*BRC8 residues are labelled in parenthesis. (residues represented as sticks are coloured as in Fig 4).

BRCA2 is important for the recruitment and assembly of RAD51 [7]. PHI–BLAST was used to find BRC repeats in *Os*BRCA2. Similar to *Hs*BRCA2, *Os*BRCA2 was found to contain 8 BRC repeats. Sequence alignment of *Hs*BRC and *Os*BRC repeats showed the presence of a conserved motif with a notable phenylalanine. *Hs*BRC repeats contained a conserved sequence FxxA. However, in *Os*BRC repeats, glycine was the predominant residue in this motif instead of alanine, i.e., FxxG (Fig 6A). To evaluate the effect of BRC repeats of *Os*BRCA2, models of *Os*RAD51 were generated separately with all 8 *Os*BRC repeats using AlphaFold. It has been reported that *Hs*BRC3 and *Hs*BRC4 bind to distinct regions of *Hs*RAD51. *Hs*BRC3 interacts with N-terminal domain whereas *Hs*BRC4 binds to the nucleotide-binding core of *Hs*RAD51 [33]. However, AlphaFold predicted all *Os*BRC repeats to bind to the same site at the nucleotide-binding core of *Os*RAD51 with the conserved FxxG/A motif occupying the protomer-protomer interface important for oligomerization of *Os*RAD51 (S3 Fig). So, based on the high sequence similarity of *Os*BRC8 to *Hs*BRC4 (and presence of FxxA motif similar to *Hs*BRC4), it was modelled with *Os*RAD51. It was found that *Os*BRC8 interact with *Os*RAD51 via both polar and hydrophobic interactions. R250 of *Os*RAD51 interact with N278 of *Os*BRC8, E213 with S256 and E187 with T248 and S246, respectively (Fig 6B i). Likewise, *Hs*BRC4 showed a similar pattern of polar interactions with *Hs*RAD51 (Fig 6B ii, PDB ID 1N0W). In addition, the non-polar residues (notably F252, A254 and L274) of *Os*BRC8 are buried into hydrophobic pockets of *Os*RAD51 (Fig 6C i) which is comparable to the non-polar interaction of *Hs*BRC4-*Hs*RAD51 (Fig 6C ii, PDB ID 1N0W). Overlay of *Os*BRC8 with *Os*RAD51 reveals that the former binds and blocks the non-polar interaction (Fig 4C) between *Os*RAD51 protomers. Fig 6D shows the conserved motif of second protomer (85-GFTSA-89, yellow) interacts with the short hydrophobic sheet (190-AYA-192, green) of the first protomer. The non-polar residues of *Os*BRC8 interact and mask this region. In particular, F252 of *Os*BRC8 resides in a cavity which is occupied by F86 of the second promoter – an interaction required for filament assembly [28]. As this phenylalanine is highly conserved in both *Hs*BRC and *Os*BRC repeats (Fig 6A), *Os*BRC repeat may lead to the disassembly of *Os*RAD51.

### Interaction with CAM833

**Fig 7:**
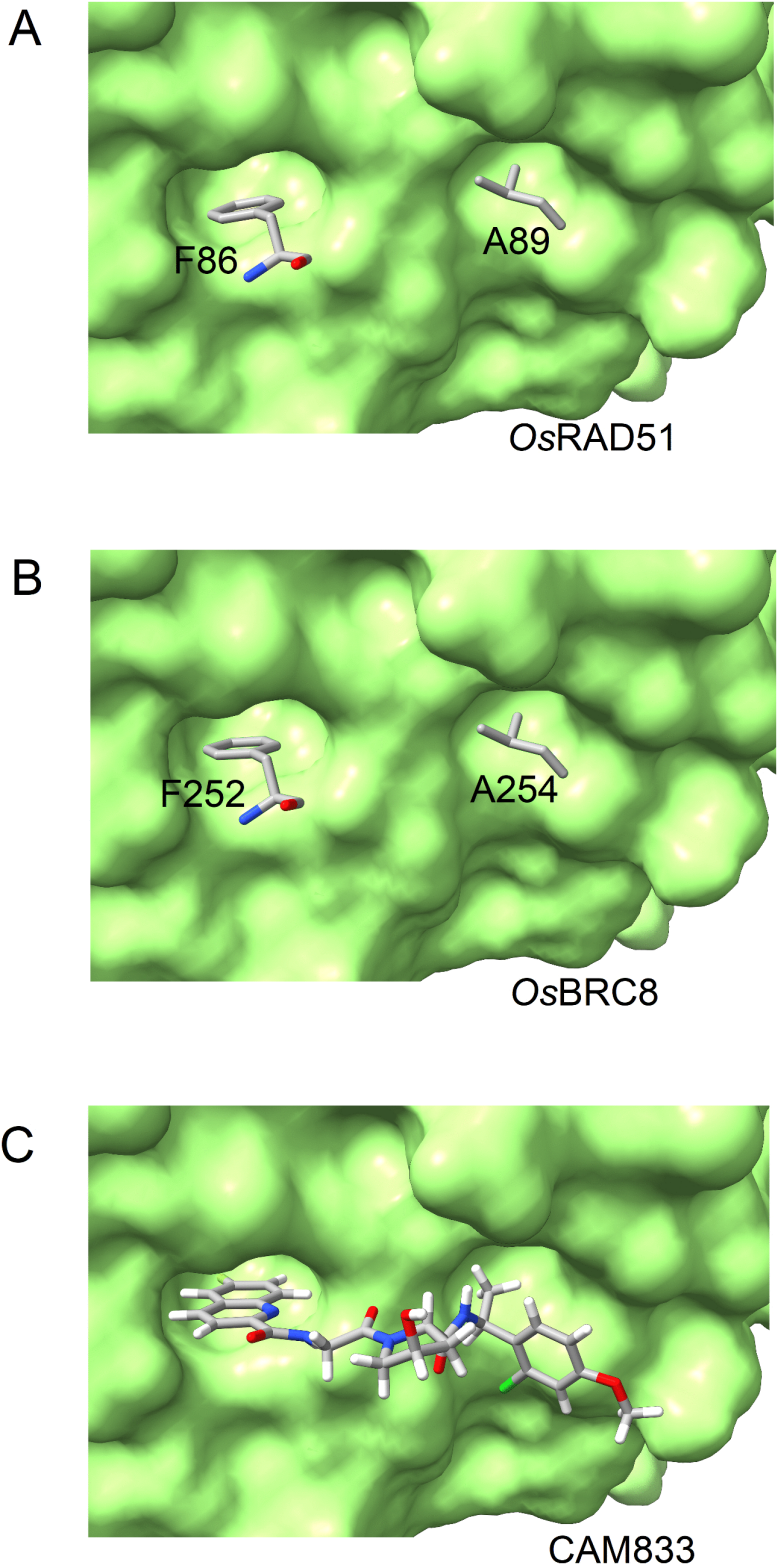
Phe and Ala binding pockets of *Os*RAD51 (green) bound by (A) F86 and A89 of *Os*RAD51, (B) F1524 and A1527 of BRC repeat and (C) CAM833.

CAM833 has been shown to occupy the FxxA-binding pocket of *Hs*RAD51 [8]. This binding may prevent the oligomerization of RAD51. To evaluate this effect, *Os*RAD51 was modelled with CAM833. It was observed that CAM833 binds the hydrophobic cavities of *Os*RAD51 required for its oligomerization via FxxA motif. During oligomerization, the Phe and Ala binding pockets of *Os*Rad51 are occupied by F86 and A89 of the second *Os*RAD51 protomer, respectively (Fig 7A). *Os*BRC8 of BRCA2 (by its FxxA motif) can bind the same hydrophobic pockets (Fig 7B) and may prevent the oligomerization of *Os*RAD51. Likewise, CAM833 may interfere with *Os*RAD51 oligomerization site by binding these Phe and Ala cavities (Fig 7C).

## Discussion

RAD51 plays a significant role in HR and DSB repair pathway. Resection of the break point is followed by RAD51 recruitment and assembly as a nucleoprotein filament which invades a homologous region and mediates recombination [5]. Moreover, RAD51 is also important in preventing transcription-replication conflicts [34], stabilizing replication fork [35] and protecting abasic sites [36]. BRCA2 is a key regulator of RAD51 assembly with BRC repeats preventing RAD51 nucleoprotein filament formation while its C-terminal domain (TR2) stabilizing RAD51 binding to double strand DNA [37].

In this study, sequence and *in silico* structural analysis of *Os*RAD51 was done. Multiple sequence alignment of *Os*RAD51 with other plant, yeast and well-studied human (*Hs*RAD51) orthologs was done. The analysis showed typical sequence features in *Os*RAD51. Walker A and Walker B motif, required for ATP-binding and hydrolysis [5], were identified in *Os*RAD51. In addition, Walker A motif also contributes to protomer-protomer interaction [26]. *Hs*RAD51 contains two conserved acidic residues (D184 and D187). These are important for binding of TR2 domain of BRCA2. TR2 stabilizes RAD51 and prevent its disassembly [28,38]. Sequence analysis showed that *Os*RAD51 contained conserved acidic residues on the corresponding positions (D184 and E187) thus indicating a similar mechanism of nucleoprotein filament stabilization.

The activity of RAD51 is regulated by PTMs including ubiquitination and phosphorylation. Covalent attachment of ubiquitin and ubiquitin-like proteins mediate many regulatory processes including protein turnover [39]. It has been reported that RAD51 ubiquitination interferes with its interaction with BRCA2 [29]. Also, p53 upregulated modulator of apoptosis (PUMA) can facilitate ubiquitination of RAD51 thus repressing HR repair [40]. Several lysine residues of RAD51 have been shown to be important for its ubiquitination including K57, K58, K64, K107 and K156 [41]. We identified six lysine residues as potential ubiquitination sites in *Os*RAD51 (Fig 2). Among these, K58, K64 and K156 were identical to those of *Hs*RAD51 as reported earlier [41]. Moreover, K57 and K107 of *Hs*RAD51 corresponded to R57 and R107 in *Os*RAD51 (Fig 1) indicating these residues as potential ubiquitination sites in *Os*RAD51. In addition to ubiquitination, RAD51 activity can also be regulated by its phosphorylation. Several putative phosphorylation sites in *Os*RAD51 were identified. Some of these reside within or adjacent to the protomer-protomer interface and ATP-binding site. Y54 of *Os*RAD51 was identified as a possible phosphorylation site. This residue forms the aromatic protomer interface and interacts with Y195 of the next protomer. So, phosphorylation of Y54 is expected to hinder this hydrophobic interaction. This is also supported by the observation that phosphorylation of the corresponding residue in *Hs*RAD51 resulted in inhibition of DNA-binding and strand exchange [42]. Another potential phosphorylation site of *Os*RAD51 is Y126 which lies adjacent to Walker A motif. Phosphorylation of the corresponding residue (Y77) of *Deinococcus radiodurans* RAD51 has been reported to increase its affinity for dsDNA [43]. S131 of *Os*RAD51 lies within the Walker A motif and is a predicted phosphorylation site. Phosphorylation of the corresponding residue (T131) of *Hs*RAD51 was shown to result in DNA-independent ATPase activity [44]. In *Hs*RAD51, the phosphorylation of T13 and S14 is important for its interaction with MRN complex [45]. However, in both multiple sequence alignment and phosphorylation-site prediction, corresponding residues were not detected in *Os*RAD51 or other plant RAD51 orthologs. So, this phosphorylation-based regulation might not occur in *Os*RAD51.

Several studies have described the structure of RAD51 nucleoprotein filament [28,46–48]. After forming a nucleoprotein filament and homologous DNA binding, RAD51 mediates strand exchange. We evaluated the formation of *Os*RAD51 nucleoprotein filament. AlphaFold was used to model *Os*RAD51 nucleoprotein filament. Conserved interacting residues of *Os*RAD51, as identified in Fig 1, were found at protomer-protomer interface (Fig 4). The protomers interacted via both polar and non-polar interactions. A conserved ATP binding site was also found at the interface of *Os*RAD51 protomers [49]. Recently, it was reported that S192 in Walker A motif of *Sc*RAD51 has an important role in ATP hydrolysis. It is suggested that the side chain of S192 could interact with ATP and together with K191 can facilitate ATP hydrolysis [50]. In *Os*RAD51 these conserved positions of Walker A motif correspond to T134 and K133, respectively. Moreover, all plant and human orthologs in Fig 1 have a conserved threonine and lysine at these positions, respectively, indicating a similar conserved mechanism of ATP hydrolysis. RAD51 interacts with bound DNA through the conserved loops L1 and L2 leading to formation of a hetroduplex structure [27,51,52]. Likewise, our model of *Os*RAD51 showed that L1 and L2 surround the DNA and mainly interact with the DNA backbone (Fig 4B). The nucleotides of bound DNA interact with *Os*RAD51 as triplets (Fig 4C). A conserved valine in L2 (V273 of *Os*RAD51) inserts between the triplets to partition them. Similarly, a recent study reported that DNA nucleotides interact with *Sc*RAD51 as triplets with a conserved V331 separating the nucleotide triplets. Moreover, R293 of *Sc*RAD51, important for twisting and unstacking of DNA bases, was found to have multiple conformations. The dynamic nature of R293 may have an important role in inserting V331 to separate the bound DNA triplets [50]. In *Os*RAD51, the corresponding residue R235 was found to occupy a similar position close to the base triplets with V273 (corresponding to *Sc*RAD51 V331) inserted between the triplets (Fig 5B).

*Hs*BRCA2 contains 8 BRC repeats which are important for the regulation of *Hs*RAD51. Among these, *Hs*BRC3 and *Hs*BRC4 have been shown to bind to distinct regions of *Hs*RAD51 [33]. However, in our AlphaFold model of *Os*RAD51, all *Os*BRC repeats were predicted to bind to the same region (S3 Fig). This is probably because of the reason that all *Os*BRC repeats contain the FxxA/G motif with some conservation in the neighbouring region as well. And in the absence of a template, AlphaFold rely on sequence alignments to generate a model. So, this may have led to modelling all *Os*BRC repeats in the same region of *Os*RAD51. Moreover, BRCA2 contains two types of motifs which can regulate RAD51 assembly. The first one, FxxA motif (A-motif), is found in BRC repeats and prevent nucleoprotein filament assembly. The second, FxPP motif (P-motif), binds and stabilizes the nucleoprotein filament [53]. The FxxA motif of *Hs*BRC repeat competes with *Hs*RAD51 and prevents its oligomerization. Similarly, *Os*BRCA2 also contains 8 BRC repeats with a conserved phenylalanine (FxxG). Presence of this motif in *Os*BRC repeats indicates that *Os*BRC repeats can regulate *Os*RAD51 assembly in a manner similar to *Hs*BRC repeats. In addition, *Hs*BRCA2 contain two FxPP motifs. This protects the nucleoprotein filament against disruption by BRC repeats. FxPP interacts with loops 148-IDRGGGE-154 and 178-YGLS-181 of *Hs*RAD51 [54]. *Os*RAD51 was found to contain corresponding sequences with conserved residues suggesting its regulation by *Os*BRCA2. This suggests that *Os*BRCA2 may contribute to both stability and disruption of *Os*RAD51 nucleoprotein filaments. Moreover, RAD51 has an intrinsic ability to oligomerize. This structural complexity makes it difficult to obtain RAD51 in monomeric form. One strategy recently reported was based on generation of a double mutant (F86E, A89E) in which the oligomerization interface is mutated and results in producing monomeric RAD51 [55]. However, oligomerization inhibitors may be used as an alternate strategy. CAM833 may inhibit oligomerization of RAD51 by occupying the F86 and A89 binding pockets [8]. To evaluate this, a model of *Os*RAD51 with CAM833 was generated. Our result suggests that CAM833 might block the oligomerization interface of *Os*RAD51 (Fig 7C). So, this approach may be useful in studying *Os*RAD51 in monomeric state. Moreover, this strategy may also be beneficial in studying HR and genetic stability in *O. sativa*. A recent report described the mechanism of strand exchange by *Hs*RAD51 and identified several residues for D-loop formation. These include F279 in loop L2 (strand separation); R303, K304, R306, K313 (DNA capture); K39, K40, K64, K70 and K73 (arm duplex binding) [56]. Sequence alignment (Fig 1) showed that all these important residues are conserved in *Os*RAD51 as well.

So, taken together, these findings suggest that *Os*RAD51 may form nucleoprotein filament and can be regulated by *Os*BRCA2. *Os*RAD51 is essential for HR and meiosis in rice signified by the observation that *Os*RAD51 mutant rice plants are sterile [13,14]. In addition to these physiological roles, the DNA binding domain (DBD) of *Os*RAD51 can used to improve DNA editing systems. Although the physiological role of *Os*RAD51 is much explored, to our knowledge, no structural investigations have been done. Our study sheds light on structure and regulation of assembly of *Os*RAD51 providing structure-function relationship.

## Supporting information

S1 Fig Pairwise alignment of *Os*Rad51 with other eukaryotic homologs. (Percent identity is shown in brackets). *Zm*Rad51, *Zea mays* RAD51, Uniprot ID: Q67EU8 (94%). *At*Rad51, *Arabidopsis thaliana* RAD51, Uniprot ID: P94102 (86%). *Sc*Rad51, *Saccharomyces cerevisiae* RAD51, Uniprot ID: P25454 (55%). *Hs*Rad51, *Homo sapiens* RAD51, Uniprot ID: Q06609 (69%).
(PDF)

S2 Fig Superposition of *Os*RAD51 monomeric models generated by AlphaFold 3 and MODELLER10.5. AlphaFold *Os*RAD51 model without using template (cyan), AlphaFold *Os*RAD51 model using template (yellow) and homology model of *Os*RAD51 by using MODELLER (green). Loops L1 and L2 are shown.
(PDF)

S3 Fig Superposition of modelled *Os*BRC repeats with *Os*RAD51 (green surface). Conserved phenylalanine and alanine/ glycine of FxxA/G motif are shown as sticks. *Os*BRC1 (red), *Os*BRC2 (yellow), *Os*BRC3 (cyan), *Os*BRC4 (magenta), *Os*BRC5 (wheat), *Os*BRC6 (grey), *Os*BRC7 (orange) and *Os*BRC8 (blue).
(PDF)

S1 Table Comparison of *Os*RAD51 models. (PDF)
