# Supplementary material for: In silico structural analysis of Oryza sativa RAD51 reveals key interactions for nucleoprotein filament assembly and regulation
Source: PLoS One. 2025 Nov 12;20(11):e0335974. doi: 10.1371/journal.pone.0335974 (PMC12611145; doi:10.1371/journal.pone.0335974)
Supplement: S1 Fig — (Percent identity is shown in brackets). ZmRad51, Zea mays RAD51, Uniprot ID: Q67EU8 (94%). AtRad51, Arabidopsis thaliana RAD51, Uniprot ID: P94102 (86%). ScRad51, Saccharomyces cerevisiae RAD51, Uniprot ID: P25454 (55%). HsRad51, Homo sapiens RAD51, Uniprot ID: Q06609 (69%). (PDF) [file pone.0335974.s001.pdf]

```
OsRad51          1 MSTSA---AAAAAAEQQQEEGEHGPFPIEQLQASGIAALDVKKLKDSGLY     47
                   ||::|   ..|||||  |||.||||||||||||||||||||||||||||:
ZmRad51          1 MSSAAQQQQKAAAAE--QEEVEHGPFPIEQLQASGIAALDVKKLKDSGLH     48

OsRad51         48 TVESVAYTPRKDLLQIKGISEAKVDKIVEAASKLVPLGFTSASQLHAQRL     97
                   |||:|||||||||||||||||||.|||:|||||:||||||||||||||||
ZmRad51         49 TVEAVAYTPRKDLLQIKGISEAKADKIIEAASKIVPLGFTSASQLHAQRL     98

OsRad51         98 EIIQVTTGSRELDKILDGGIETGSITEIYGEFRSGKTQLCHTLCVTCQLP    147
                   ||||||||||||||||:|||||||||||||||||||||||||||||||||
ZmRad51         99 EIIQVTTGSRELDKILEGGIETGSITEIYGEFRSGKTQLCHTLCVTCQLP    148

OsRad51        148 LDQGGGEGKALYIDAEGTFRPQRLLQIADRFGLNGADVLENVAYARAYNT    197
                   ||||||||||||||||||||||||||||||||||||||||||||||||||
ZmRad51        149 LDQGGGEGKALYIDAEGTFRPQRLLQIADRFGLNGADVLENVAYARAYNT    198

OsRad51        198 DHQSRLLLEAASMMIETRFALMIVDSATALYRTDFSGRGELSARQMHMAK    247
                   ||||||||||||||||||||||:|||||||||||||||||||||||||||
ZmRad51        199 DHQSRLLLEAASMMIETRFALMVVDSATALYRTDFSGRGELSARQMHMAK    248

OsRad51        248 FLRSLQKLADEFGVAVVITNQVVAQVDGSAMFAGPQIKPIGGNIMAHAST    297
                   ||||||||||||||||||||||||||||||||||||.|||||||||||||
ZmRad51        249 FLRSLQKLADEFGVAVVITNQVVAQVDGSAMFAGPQFKPIGGNIMAHAST    298

OsRad51        298 TRLALRKGRGEERICKVISSPCLAEAEARFQIASEGVADVKD    339
                   |||||||||||||||||||||||||||||||:||||:|||||
ZmRad51        299 TRLALRKGRGEERICKVISSPCLAEAEARFQLASEGIADVKD    340
```

```
OsRad51            1 ---MSTSAAAAAAAEQQQEEGEHGPFPIEQLQASGIAALDVKKLKDSGLY     47
                        |.......|..:|..||.:|||||:|||||:|||::|||||:|:||.
AtRad51            1 MTTMEQRRNQNAVQQQDDEETQHGPFPVEQLQAAGIASVDVKKLRDAGLC     50

OsRad51           48 TVESVAYTPRKDLLQIKGISEAKVDKIVEAASKLVPLGFTSASQLHAQRL     97
                     |||.||||||||||||||||:||||||||||||||||||||||||||||.
AtRad51           51 TVEGVAYTPRKDLLQIKGISDAKVDKIVEAASKLVPLGFTSASQLHAQRQ    100

OsRad51           98 EIIQVTTGSRELDKILDGGIETGSITEIYGEFRSGKTQLCHTLCVTCQLP    147
                     ||||:|:|||||||:|:||||||||||:||||||||||||||||||||||
AtRad51          101 EIIQITSGSRELDKVLEGGIETGSITELYGEFRSGKTQLCHTLCVTCQLP    150

OsRad51          148 LDQGGGEGKALYIDAEGTFRPQRLLQIADRFGLNGADVLENVAYARAYNT    197
                     :|||||||||:||.||||||||||||||||||||||||||||||||||||
AtRad51          151 MDQGGGEGKAMYIGAEGTFRPQRLLQIADRFGLNGADVLENVAYARAYNT    200

OsRad51          198 DHQSRLLLEAASMMIETRFALMIVDSATALYRTDFSGRGELSARQMHMAK    247
                     ||||||||||||||||||||||:||||||||||||||||||||||||:||
AtRad51          201 DHQSRLLLEAASMMIETRFALLIVDSATALYRTDFSGRGELSARQMHLAK    250

OsRad51          248 FLRSLQKLADEFGVAVVITNQVVAQVDGSAMFAGPQIKPIGGNIMAHAST    297
                     ||||||||||||||||||||||||||||||:|||||.|||||||||||:|
AtRad51          251 FLRSLQKLADEFGVAVVITNQVVAQVDGSALFAGPQFKPIGGNIMAHATT    300

OsRad51          298 TRLALRKGRGEERICKVISSPCLAEAEARFQIASEGVADVKD    339
                     |||||||||.|||||||||||||.||||||||::|||.|.||
AtRad51          301 TRLALRKGRAEERICKVISSPCLPEAEARFQISTEGVTDCKD    342
```

```
OsRad51              1 -----------------------------------------------MST      3
                                                                      .:.
ScRad51              1 MSQVQEQHISESQLQYGNGSLMSTVPADLSQSVVDGNGNGSSEDIEATNG     50

OsRad51              4 SAAAAAAAEQQQEEGEH----------GPF-PIEQLQASGIAALDVKKLK     42
                       |.......||.:.:||.          |.| |||:||.:||...|||||:
ScRad51             51 SGDGGGLQEQAEAQGEMEDEAYDEAALGSFVPIEKLQVNGITMADVKKLR    100

OsRad51             43 DSGLYTVESVAYTPRKDLLQIKGISEAKVDKIVEAASKLVPLGFTSASQL     92
                       :|||:|.|:|||.||||||:||||||||.||::..|::|||:||.:|:..
ScRad51            101 ESGLHTAEAVAYAPRKDLLEIKGISEAKADKLLNEAARLVPMGFVTAADF    150

OsRad51             93 HAQRLEIIQVTTGSRELDKILDGGIETGSITEIYGEFRSGKTQLCHTLCV    142
                       |.:|.|:|.:||||:.||.:|.||:|||||||::||||:||:||||||.|
ScRad51            151 HMRRSELICLTTGSKNLDTLLGGGVETGSITELFGEFRTGKSQLCHTLAV    200

OsRad51            143 TCQLPLDQGGGEGKALYIDAEGTFRPQRLLQIADRFGLNGADVLENVAYA    192
                       |||:|||.||||||.||||.||||||.||:.||.||||:..|.|.|||||
ScRad51            201 TCQIPLDIGGGEGKCLYIDTEGTFRPVRLVSIAQRFGLDPDDALNNVAYA    250

OsRad51            193 RAYNTDHQSRLLLEAASMMIETRFALMIVDSATALYRTDFSGRGELSARQ    242
                       ||||.|||.|||..||.||.|:||:|::|||..|||||||||||||||||
ScRad51            251 RAYNADHQLRLLDAAAQMMSESRFSLIVVDSVMALYRTDFSGRGELSARQ    300

OsRad51            243 MHMAKFLRSLQKLADEFGVAVVITNQVVAQVDGSAMFAGPQIKPIGGNIM    292
                       ||:|||:|:||:|||:||||||:||||||||||...|.....||||||||
ScRad51            301 MHLAKFMRALQRLADQFGVAVVVTNQVVAQVDGGMAFNPDPKKPIGGNIM    350

OsRad51            293 AHASTTRLALRKGRGEERICKVISSPCLAEAEARFQIASEGVADVKD---    339
                       ||:|||||..:||:|.:|:|||:.||||.|||..|.|..:||.|.::
ScRad51            351 AHSSTTRLGFKKGKGCQRLCKVVDSPCLPEAECVFAIYEDGVGDPREEDE    400
```

```
OsRad51            1 MSTSAAAAAAAEQQQEEGEHGPFPIEQLQASGIAALDVKKLKDSGLYTVE     50
                     |:......|.|:...||...||.||.:|:..||.|.|||||:::|.:|||
HsRad51            1 MAMQMQLEANADTSVEEESFGPQPISRLEQCGINANDVKKLEEAGFHTVE     50

OsRad51           51 SVAYTPRKDLLQIKGISEAKVDKIVEAASKLVPLGFTSASQLHAQRLEII    100
                     :|||.|:|:|:.||||||||.|||:..|:||||:|||:|::.|.:|.|||
HsRad51           51 AVAYAPKKELINIKGISEAKADKILAEAAKLVPMGFTTATEFHQRRSEII    100

OsRad51          101 QVTTGSRELDKILDGGIETGSITEIYGEFRSGKTQLCHTLCVTCQLPLDQ    150
                     |:||||:||||:|.||||||||||::||||:||||:||||.||||||:|:
HsRad51          101 QITTGSKELDKLLQGGIETGSITEMFGEFRTGKTQICHTLAVTCQLPIDR    150

OsRad51          151 GGGEGKALYIDAEGTFRPQRLLQIADRFGLNGADVLENVAYARAYNTDHQ    200
                     |||||||:|||.||||||:|||.:|:|:||:|:|||:|||||||:|||||
HsRad51          151 GGGEGKAMYIDTEGTFRPERLLAVAERYGLSGSDVLDNVAYARAFNTDHQ    200

OsRad51          201 SRLLLEAASMMIETRFALMIVDSATALYRTDFSGRGELSARQMHMAKFLR    250
                     ::||.:|::||:|:|:||:||||||||||||:||||||||||||:|:|||
HsRad51          201 TQLLYQASAMMVESRYALLIVDSATALYRTDYSGRGELSARQMHLARFLR    250

OsRad51          251 SLQKLADEFGVAVVITNQVVAQVDGSAMFAGPQIKPIGGNIMAHASTTRL    300
                     .|.:|||||||||||||||||||||:||||....|||||||:||||||||
HsRad51          251 MLLRLADEFGVAVVITNQVVAQVDGAAMFAADPKKPIGGNIIAHASTTRL    300

OsRad51          301 ALRKGRGEERICKVISSPCLAEAEARFQIASEGVADVKD    339
                     .|||||||.||||:..||||.||||.|.|.::||.|.||
HsRad51          301 YLRKGRGETRICKIYDSPCLPEAEAMFAINADGVGDAKD    339
```

S1 Fig: Pairwise alignment of *Os*Rad51 with other eukaryotic homologs.
