# Supplementary figures and images for: In silico structural analysis of Oryza sativa RAD51 reveals key interactions for nucleoprotein filament assembly and regulation
Source: PLoS One. 2025 Nov 12;20(11):e0335974. doi: 10.1371/journal.pone.0335974 (PMC12611145; doi:10.1371/journal.pone.0335974)

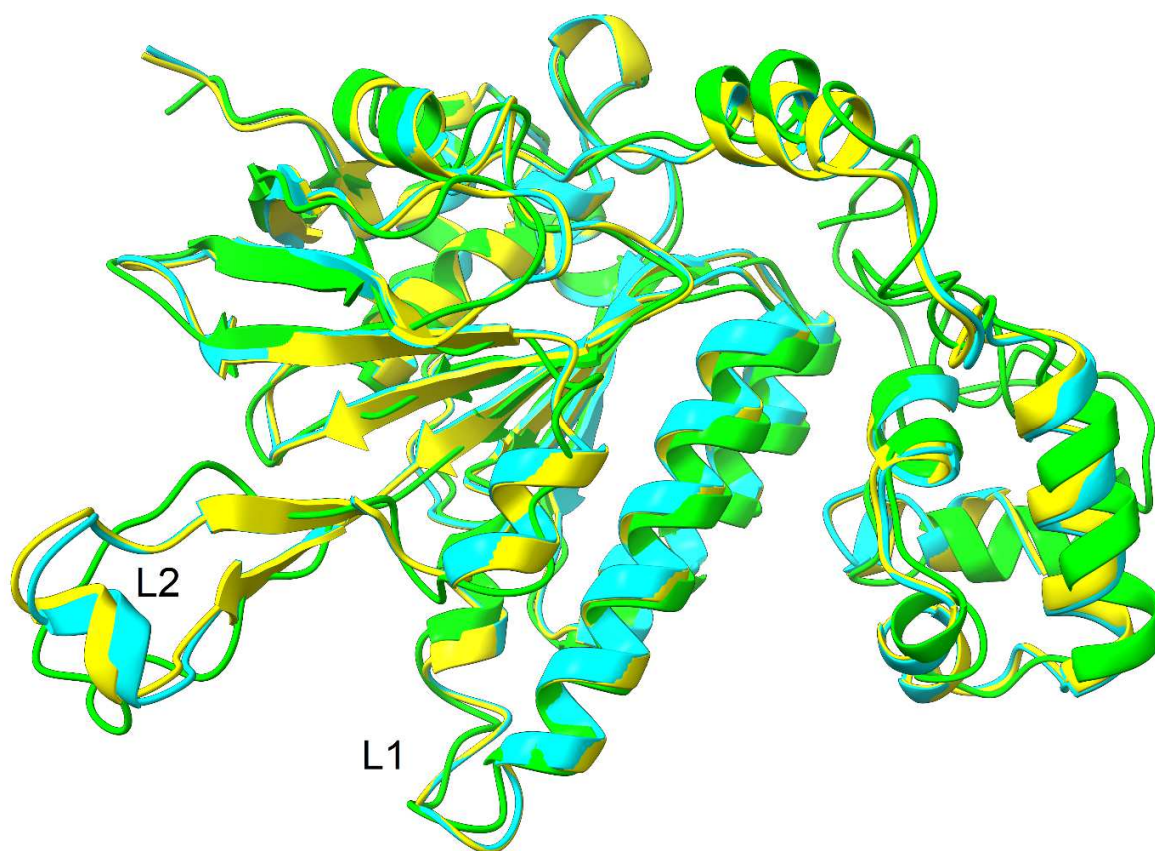

S2 Fig: Superposition of *OsRAD51* monomeric models generated by AlphaFold 3 and MODELLER10.5.

Supplement: S2 Fig — AlphaFold OsRAD51 model without using template (cyan), AlphaFold OsRAD51 model using template (yellow) and homology model of OsRAD51 by using MODELLER (green). Loops L1 and L2 are shown. (PDF) [file pone.0335974.s002.pdf]

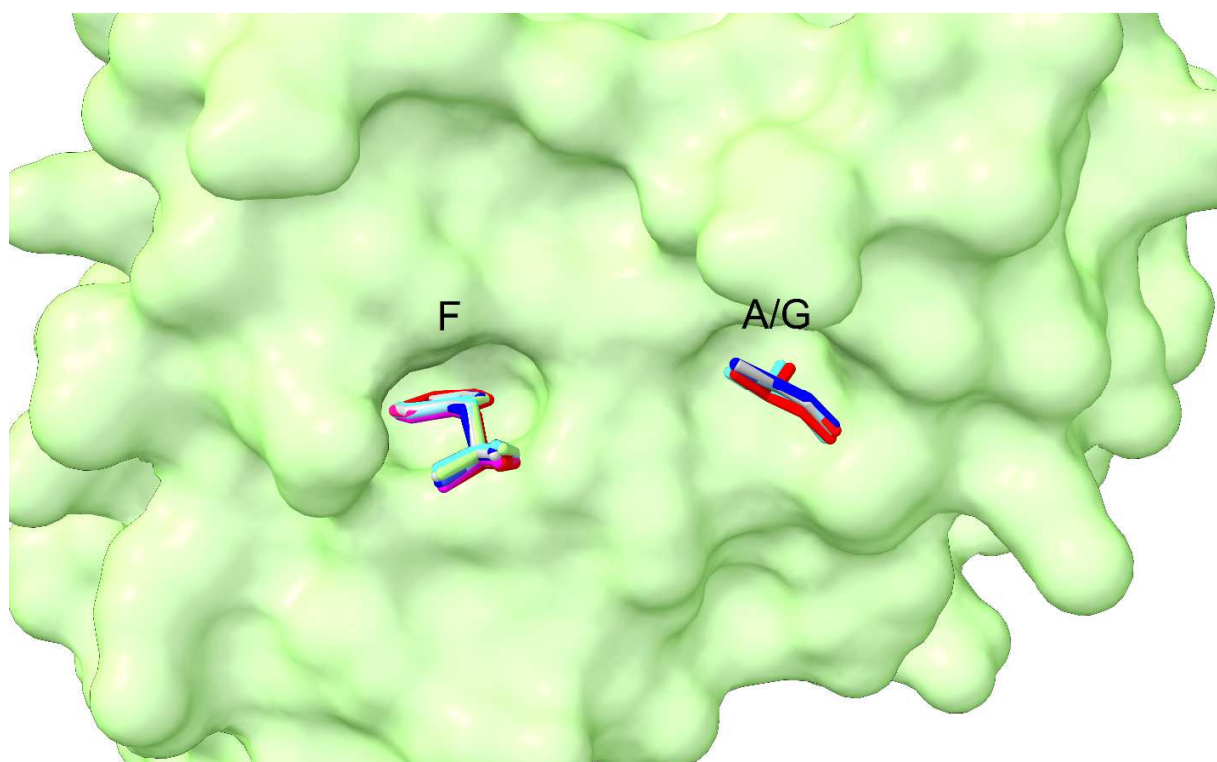

S3 Fig: Superposition of modelled *OsBRC* repeats with *OsRAD51* (green surface).

Supplement: S3 Fig — Conserved phenylalanine and alanine/ glycine of FxxA/G motif are shown as sticks. OsBRC1 (red), OsBRC2 (yellow), OsBRC3 (cyan), OsBRC4 (magenta), OsBRC5 (wheat), OsBRC6 (grey), OsBRC7 (orange) and OsBRC8 (blue). (PDF) [file pone.0335974.s003.pdf]
