# Supplementary material for: In silico structural analysis of Oryza sativa RAD51 reveals key interactions for nucleoprotein filament assembly and regulation
Source: PLoS One. 2025 Nov 12;20(11):e0335974. doi: 10.1371/journal.pone.0335974 (PMC12611145; doi:10.1371/journal.pone.0335974)
Supplement: S1 Table — (PDF) [file pone.0335974.s004.pdf]

S1 Table: Comparison of *Os*RAD51 models

Model Name	AlphaFold (without template)	AlphaFold (using PDB template)	MODELLER
Template	None	*Hs*RAD51 (PDB ID 5JZC)	HsRAD51 (PDB ID 5H1B)
	Backbone RMSD		
AlphaFold (without template)	0	0.075	1.03
AlphaFold (using PDB template)	0.075	0	1.06
MODELLER	1.03	1.06	0
